# Novel uterine sarcoma preoperative diagnosis score predicts the need for surgery in patients presenting with a uterine mass

**DOI:** 10.1186/2193-1801-3-678

**Published:** 2014-11-18

**Authors:** Tomonori Nagai, Yasushi Takai, Taichi Akahori, Hiroaki Ishida, Tatsuya Hanaoka, Takahiro Uotani, Sho Sato, Shigetaka Matsunaga, Kazunori Baba, Hiroyuki Seki

**Affiliations:** Department of Obstetrics and Gynecology, Saitama Medical Center, Saitama Medical University, 1981 Kamoda, Kawagoe-shi, Saitama, Japan

**Keywords:** Uterine sarcoma, Preoperative diagnosis, Multiple predictors

## Abstract

Preoperative diagnosis of uterine sarcoma is very difficult, and currently, its diagnostic accuracy is not satisfactory. It is therefore important to perform surgery and establish the pathological diagnosis if the clinical findings and various examination findings indicate possible uterine sarcoma. We investigated the accuracy of the combination of various types of predictors of uterine sarcoma and the novel **PRE**operative **S**arcoma **S**core (PRESS) for avoiding unnecessary surgery while diagnosing uterine sarcoma.

We retrospectively analyzed the clinical findings, blood tests, imaging studies (ultrasonography and magnetic resonance imaging [MRI]), and endometrial cytology of 63 suspected uterine sarcoma cases that underwent surgery from 2006 to 2012. These cases were also scored retrospectively using PRESS. We analyzed the number of unnecessary surgeries that could be avoided using PRESS.

Of 63 cases, 15 were diagnosed with uterine sarcoma (sarcoma group), and 48 had benign tumors (benign group). Univariate analysis indicated age, serum lactate dehydrogenase (LDH) values, and MRI and endometrial cytology findings as significant predictors of uterine sarcoma in both groups. In contrast, multivariable analysis identified only age, serum LDH value, and endometrial cytology findings as significant predictors. Accordingly, the latter were placed as 2 points, and the remaining MRI finding as 1 point. The accuracy rate of prediction was 84.1%, and the positive and negative predictive values were 63.2% and 93.2% respectively when the PRESS was interpreted as “positive” when it was 3 points or higher.

Using multiple predictors for the preoperative diagnosis of uterine sarcoma, our proposed PRESS score is beneficial in the clinical setting while making treatment decisions in suspected uterine sarcoma cases as well as avoiding unnecessary surgery.

## Background

Uterine sarcoma is a rare disease, accounting for 3–5% of all malignancies of the uterine body. In the United States, its incidence in African-Americans is twice that of Caucasian women (Trope et al. 2012). Because hematogenous metastasis often occurs early and the disease is often resistant to radiation and chemotherapy, the prognosis is poor without early surgical intervention (Gadducci et al. 2008). Patients with a rapidly enlarging uterine tumors, uterine tumors and abnormal vaginal bleeding, or giant uterine tumors are suspected to have uterine sarcomas. In such cases, a multilateral evaluation is conducted for uterine tumor.

Uterine sarcoma presents as a heterogeneous uterine mass on ultrasonography. Color Doppler imaging shows a low tumor blood flow resistance index (RI) (Szabo et al. 2002; Kurjak et al. 1995; Aviram et al. 2005). On MRI, high intratumoral signal intensity on T1-weighted images (intratumoral hemorrhage and coagulative necrosis) and a heterogeneous mass on T2 weighted images are typical findings of uterine sarcoma (Kido et al. 2003; Sahdev et al. 2001). Elevated lactate dehydrogenase (LDH) and cancer antigen 125 (CA 125) levels are useful biomarkers for preoperative diagnosis (Seki et al. 1992; Juang et al. 2006; Goto et al. 2002). Abnormal endometrial cytology may also be seen if uterine sarcoma protrudes into the uterine cavity (Oda et al. 2004; Ito et al. 2004). However, these findings are not sufficiently accurate for making a preoperative diagnosis of uterine sarcoma. In practice, hysterectomy and histopathological examination are necessary in order to differentiate uterine sarcoma from uterine myoma, the most common gynecologic tumor (Nam 2011).

To date, no scoring system with multiple predictors has been described for the preoperative diagnosis of uterine sarcoma. In this study, we retrospectively analyzed the preoperative clinical, laboratory, and imaging findings in patients who underwent surgery for suspected uterine sarcoma. We combined various predictors considered useful for the preoperative diagnosis of this disease and developed a score: **PRE**operative **S**arcoma **S**core: PRESS. We also analyzed the degree of diagnostic accuracy of PRESS and retrospectively determined its predictive value to prevent unnecessary surgery.

## Patients and methods

All patients who underwent surgery at Saitama Medical Center, Saitama Medical University from January 2006 to December 2012 for suspected uterine sarcoma were retrospectively evaluated. Study parameters included age at the time of surgery, clinical findings, blood test results, imaging study (specifically ultrasonography and magnetic resonance imaging [MRI]) and endometrial cytology findings, and postoperative pathological diagnosis. This study complies with the guidelines specified in the Declaration of Helsinki and Japanese ethical guidelines for observational studies. Therefore this study was approved by Institutional Review Board (IRB) of Saitama Medical Center, Saitama Medical University without the necessity to obtain written informed consent from the patients for the publication of this report.

Clinical findings were defined as patients’ complaints at admission to hospital, including abnormal vaginal bleeding, bloating, and the rapid enlargement of uterine myoma. The maximum diameter of the uterine tumor was measured using MRI (in cm). Blood was obtained for preoperative evaluation of serum CA125 and LDH levels. Ultrasonographic evaluation of all cases was performed by the same ultrasound specialist (K.B.), and the findings were included morphologic study of the tumor and Doppler measurement of intratumoral blood flow. Doppler measurement of intratumoral RI was obtained in several places and the lowest value was recorded, since there is an inverse relationship between RI value and probability of malignancy. Data reported by Kurjak et al. (Kurjak et al. 1995) showed a sensitivity of 90.91% and specificity of 99.82% when an intratumoral blood flow RI value of 0.4 was used as a cut-off point; accordingly, we considered RI below 0.4 as positive for uterine sarcoma. On preoperative MRI, intratumoral hyperintense signal on T1-weighted images and/or a heterogeneous signal found on T2-weighted images were considered indication of uterine sarcoma. On preoperative endometrial cytology examination, abnormalities of class III or greater were considered positive for uterine sarcoma. Class III is defined as the stage when cells may show cellular or structural atypia but are not malignant cell.

Statistical analysis on the collected data was performed using Student’s *t* test and chi-square test. Welch’s *t* test for non-parametric data was used instead of Student’s *t* test for LDH on the basis of the F-test results. Appropriate cut-off points for age, serum LDH value, and PRESS were obtained using receiver operating characteristic (ROC) curves corresponding to the point on the curve nearest to the upper left corner of the ROC graph. Significant results were subjected to multivariate logistic regression analysis. Statistical significance was set at P <0.05. JMP version 10 software was used for statistical analysis.

## Results

**Figure 1:**
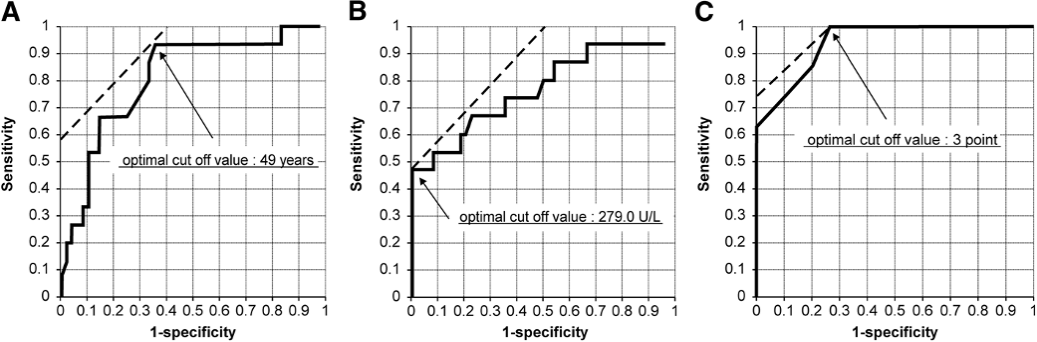
**ROC curve analysis for age, serum LDH value, and PRESS.** **A**: ROC curve of age; **B**: ROC curve of serum LDH levels; **C**: ROC curve of PRESS. The optimum cut-off point based on ROC curve analysis for age, serum LDH value, and PRESS, was 49 years **(A)**, 279.0 U/L **(B)**, and 3 points **(C)** respectively.

A total of 63 patients with suspected uterine sarcoma underwent surgery during the study period. In 15 cases (23.8%), the diagnosis of uterine sarcoma was confirmed by pathological examination (Tables 1 and 2). The mean age of all patients was 50.1 years (standard deviation [SD] 12.1, range 24–84 years). The mean age of uterine sarcoma patients (sarcoma group) was 60.3 years ([SD] 11.5, range 38–84 years), while that of patients with benign tumors (benign group) was 46.9 years ([SD] 10.4, range 24–73 years). This difference was significant (p <0.001), and the optimum cut-off point for age, based on ROC curve analysis, was 49 years (Figure 1A). About 60% of the sarcoma group patients were diagnosed with leiomyosarcoma. The final pathological diagnosis is shown in Table 2.

**Table 1:** **Patient characteristics and clinical findings**

	Total		Sarcoma		Benign		***p-value (sar. vs. ben.)***
Number of patients	63	(100%)	15	(23.8%)	48	(76.2%)	
Age (yr)	50.1	(24–84)	60.3	(38–84)	46.9	(24–73)	<0.001*
Tumor size (cm)	10.9	(3.0-23.0)	10.5	(4.9-17.0)	11.0	(3.0-23.0)	0.684
Serum CA125 (U/mL)	41.5	(3–347)	77.5	(8–347)	29.5	(3–235)	0.078
Serum LDH (U/L)	224.0	(121–941)	340.3	(134–941)	187.7	(121–255)	0.020*
RI	0.39	(0.17-0.83)	0.42	(0.23-0.83)	0.39	(0.17-0.70)	0.432
***Clinical findings***							
Rapid growth of the uterine tumor	17	(27.0%)	1	(6.7%)	16	(33.3%)	0.042*
Abnormal vaginal bleeding	14	(22.2%)	6	(40.0%)	8	(16.7%)	0.058
A palpable pelvic mass	10	(15.9%)	3	(20.0%)	7	(14.6%)	0.616
Pointed out the uterine tumor	9	(14.3%)	1	(6.7%)	8	(16.7%)	0.334
Lower abdominal pain	6	(9.5%)	2	(13.3%)	4	(8.3%)	0.565
Hypermenorrhea	5	(7.9%)	1	(6.7%)	4	(8.3%)	0.835
Cough	1	(1.6%)	1	(6.7%)	0	(0%)	0.071
Frequent urination	1	(1.6%)	0	(0%)	1	(2.1%)	0.573
Total	63	(100%)	15	(100%)	48	(100%)	

*P <0.05.

**Table 2:** **Histological analysis**

	Sarcoma		Benign	
Pathological diagnosis	Leiomyosarcoma	9 (60.0%)	Cellular leiomyoma	4 (8.3%)
	Adenosarcoma	3 (20.0%)	Leiomyoma	42 (87.5%)
	ESS	3 (20.0%)	Adenomyosis	2 (4.2%)
Total: 63 cases		15 (100%)		48 (100%)

Preoperative clinical findings are shown in Table 1. Rapid growth of the uterine tumor, a generally accepted clinical indicator of uterine sarcoma, was found in 17 cases (27.0%). However, this rapid growth of tumor was significantly more common in benign cases than in sarcoma cases (16 of 48 vs. 1 of 15; p = 0.042). In contrast, abnormal vaginal bleeding was seen in 6 sarcoma cases, and was more common in the sarcoma group than in the benign group, which did not reach statistical significance (p = 0.058).

No significant differences in maximum tumor diameters, serum CA125 values, or ultrasound RI values were observed between the two groups (Table 1). The sarcoma group had a significantly elevated mean serum LDH value (p =0.020). The optimum cut-off value, based on ROC curve analysis, was 279.0 U/L (Figure 1B). Using this cut-off value, the sensitivity of serum LDH was 0.47 and the specificity was 1.0 (p <0.001, Table 3). Positive MRI findings occurred more frequently in the sarcoma group (p <0.001), with a sensitivity and specificity of 0.80 and 0.69, respectively (Table 3). While the sensitivity of abnormal cytology findings was low (0.33), the specificity was high (0.93), and abnormal findings were observed in a significantly greater number of cases in the sarcoma group (p <0.001, Table 3).

**Table 3:** **Positive findings in patients and multivariate analysis of statistically significant positive findings**

	Total	Sarcoma	Benign	***p-value***(sar. vs. ben.)	Sensitivity	Specificity	PPV (%)	NPV (%)
Age (≥49)	31/63 (49.2%)	14/15 (93.3%)	17/48 (35.4%)	<0.001*	0.93	0.65	45.2	96.9
Serum CA125 (≥35)	17/63 (27.0%)	6/15 (40.0%)	11/48 (22.9%)	0.193	0.40	0.77	35.3	80.4
Serum LDH (≥279)	7 /63(11.1%)	7/15 (46.7%)	0/48 (0.0%)	<0.001*	0.47	1.00	100.0	85.7
RI (≤0.4)	28/63 (44.4%)	7/15 (46.7%)	29/48 (60.4%)	0.348	0.47	0.40	19.4	70.4
MRI findings	27/63 (42.9%)	12/15 (80.0%)	15/48 (31.3%)	<0.001*	0.80	0.69	44.4	91.7
Cytological findings	6/63 (9.5%)	5/15 (33.37%)	1/48 (2.1%)	<0.001*	0.33	0.93	83.3	58.3
<multivariate analysis>	odds ratio	95% CI	*p-value*					
Age	7.001×10^7^	[3.700, >1000]	0.003*					
Serum LDH	2.304×10^9^	[15.775, >1000]	<0.001*					
MRI findings	0.420	[0.014, 4.604]	0.499					
Cytological findings	4.784×10^8^	[19.554, >1000]	<0.001*					

*P <0.05.

Using the likelihood ratio test, multivariate logistic regression analysis was carried out in both groups on the significant findings, including age, rapid growth of the tumor, serum LDH values, MRI findings, and cytological findings. Age, serum LDH values, and cytological findings were found to be the significant predictors (Table 3).

The **PRE**operative **S**arcoma **S**core (PRESS) consisted of a maximum total of 7 points, 1 point for positive MRI findings and 2 points each for age, serum LDH values, and cytological findings, since the latters were strong predictors with significant results even in multivariate regression analysis (Table 4). PRESS scoring of the 63 cases revealed a mean score of 4.267 in the sarcoma group and 1.159 in the benign group (p <0.001, Table 5). Accuracy based on PRESS is shown in Table 5. The optimum cut-off value of PRESS using ROC curve was 3 points (Figure 1C). When the PRESS was interpreted as “positive” when it was 3 points or higher, the diagnostic accuracy, positive predictive value (PPV), negative predictive value (NPV), sensitivity and specificity were 84.1%, 63.2%, 93.2%, 0.800 and 0.854, respectively.

**Table 4:** **PREoperative Sarcoma Scoring system (PRESS)**

Predictors	0 point	1 point	2 points
Age	<49		≥49
Serum LDH value	<279		≥279
Cytologic findings	negative		positive
MRI findings	negative	positive	
			Total: 7 points

**Table 5:** **PREoperative Sarcoma Score (PRESS) and accuracy of PRESS for all patients**

Scoring (point)	0	1	2	3	4	5	6	7	Total	Mean score (range)
Sarcoma	0	0	3	1	0	11	0	0	15	4.267 (2–5)
Benign	22	8	11	7	0	0	0	0	48	1.159 (0–3)
Total	22	8	14	8	0	11	0	0	63	1.825 (0–8)
										p <0.001 (sar. vs. ben.)
Scoring (point)	0	≥1	≥2	≥3	≥4	≥5				
Sarcoma	0	15	15	12	11	11				
Benign	22	26	18	7	0	0				
Total	22	41	33	19	11	11				
Accuracy (%)	41.3	58.7	71.4	84.1	93.7	93.7				
PPV (%)	0.0	36.6	45.5	63.2	100.0	100.0				
NPV (%)	63.4	100.0	100.0	93.2	92.3	92.3				
Sensitivity	0	1	1	0.800	0.733	0.733				
Specificity	0.542	0.458	0.625	0.854	1	1				

## Discussion

Uterine sarcoma comprises 3–5% of all malignancies of uterine body, with a worldwide annual occurrence rate of 1.55–1.95 per 100,000 population (Trope et al. 2012). Nordal et al. (Nordal and Thoresen 1997) found that leiomyosarcoma was the most common histological tumor type (41%), followed by carcinosarcoma (35%), and endometrial stromal sarcoma (ESS) (16%). In addition, after the classification of advanced stages (FIGO 2008) was revised in 2009 by the International Federation of Gynecology and Obstetrics (FIGO), carcinosarcomas are classified as endometrial cancers, and are no longer considered uterine sarcomas (Prat 2009).

In the present study, the mean age of the 15 uterine sarcoma patients (60.3 years) was demonstrated to be an independent significant risk factor, even using multivariate regression analysis. Various studies have reported on the age of onset of uterine sarcomas, including those stating that it mostly occurs in premenopausal women at approximately 50 years of age (Montague et al. 1965) and those stating that it rather occurs mostly in postmenopausal women (Aviram et al. 2005; Parker et al. 1994); however, in an analysis of 419 uterine sarcoma cases in Norway (Abeler et al. 2009), Abeler et al. stated that the median age was 50.7 years for ESS, 56.5 years for leiomyosarcomas, and 65.7 years for adenosarcomas. We observed 3 premenopausal cases in the sarcoma group, a 38-year old woman with adenosarcoma, a 49-year old woman with leiomyosarcoma and a 51-year old woman with leiomyosarcoma. The remaining 12 cases in the sarcoma group (80.0%) were postmenopausal women.

Uterine sarcoma is much rarer than uterine myoma, the most common uterine tumor. In the pathological study of 13,000 uterine myoma cases in which surgery was performed, 38 cases (0.29%) were of leiomyosarcoma (Montague et al. 1965). Although surgery is not indicated for asymptomatic uterine myoma, hysterectomy is often considered for rapidly enlarging uterine tumors because it can be difficult to differentiate a uterine sarcoma from a uterine myoma. Current evidence, however, provides no justification for such treatment policy (Abeler et al. 2009; Parker 2007). In our 63 cases, 17 showed rapid tumor growth, but this rapid growth was actually seen in only 1 of 15 sarcoma cases, and was significantly more common in benign cases. There was no significant difference in tumor diameter between uterine sarcoma and benign tumor groups. In contrast, the incidence of abnormal vaginal bleeding was higher in the sarcoma group and was the most common clinical symptom of uterine sarcoma in our study. This supports the findings of Giuntoli et al. (Giuntoli et al. 2003) who reported 208 cases of leiomyosarcoma, of which 117 (56%) presented with abnormal vaginal bleeding.

On routine examination, all cases of tumor resembling uterine myoma should be considered as uterine sarcomas. If sarcoma is even remotely suspected from ultrasonography or clinical findings, it is important to conduct a multilateral evaluation. However, the sensitivity and specificity of various diagnostic tools, such as imaging study and tumor markers, are not presently satisfactory for differentiating benign from malignant disease.

Neither serum CA125 values nor Doppler calculated RI value was diagnostically useful in our present study. Serum CA125 values have previously been proposed as a useful preoperative diagnostic tool for uterine sarcoma (Juang et al. 2006; Patsner and Mann 1988). Juang et al. (Juang et al. 2006) analyzed 42 cases with uterine leiomyosarcoma and 84 cases with uterine leiomyoma and found that preoperative CA125 was significantly higher in uterine leiomyosarcoma. They also found that the level of this marker was higher in premenopausal women, with a cut-off point of 162 U/mL in premenopausal women vs. 75 U/mL in postmenopausal women. Several reports have evaluated the usefulness of RI value (Szabo et al. 2002; Kurjak et al. 1995; Aviram et al. 2005). In a study by Aviram et al. (Aviram et al. 2005), RI value was not significantly different between leiomyoma (n = 98) and leiomyosarcoma (n = 6). However the RI value of carcinosarcoma (n = 7) was significantly lower than that of leiomyoma. In contrast, in an analysis of 12 cases with uterine sarcoma and 117 cases with uterine leiomyoma, Szabo et al. (Szabo et al. 2002) suggested that large tumor size and necrotic degenerative and inflammatory changes were occasionally associated with lower RI value in leiomyomas as well as malignant tumors. Compared with the results of these previous studies on preoperative CA125 level and usefulness of ultrasonic Doppler RI, our study had a smaller number of subjects. We would like to re-analyze the usefulness of serum CA125 value and RI value in a larger study population in the future.

In cases of uterine sarcoma, intratumoral hyperintense signal on T2-weighted MRI images suggests high cellularity or high vascularity. In addition, hyperintense signal on T1-weighted images is thought to indicate intratumoral hemorrhage and coagulative necrosis. Goto et al. (Goto et al. 2002) found that preoperative MRI had a diagnostic accuracy of 0.971, with a sensitivity of 1.0 and specificity of 0.969 in 227 cases of uterine tumors, 10 of which were leiomyosarcoma. Our diagnostic accuracy was lower than that reported by Goto et al.; this could be because of a significant difference in the accuracy rates between the sarcoma group and benign group in our study. In addition, the study reported by Goto et al. had performed Gadolinium Diethylenetriaminepenta-acetic Acid (Gd-DTPA) -enhanced MRI; but in our study, contrast imaging using Gd-DTPA was not performed. Goto et al. also mentioned the usefulness of dynamic MRI using Gd-DTPA. Further, diffusion-weighted imaging MRI has recently been reported to have a higher preoperative diagnostic accuracy for uterine sarcoma (Tirumani et al. 2012). In the future, we hope to consider the introduction of Gd-DTPA contrast-enhanced dynamic MRI and diffusion-weighted imaging MRI in order to improve the diagnostic accuracy of MRI.

In recent years, fluorine-18-fluorodeoxyglucose positron emission tomography (FDG-PET) has been used for the diagnosis of various types of malignant tumors. Thus far, numerous studies have reported on the usefulness of FDG-PET in uterine sarcomas; however, most of them consisted of reports on the usefulness of FDG-PET in the diagnosis of recurrence of uterine sarcomas; few reports have focused on its usefulness as a means for preoperative diagnosis of uterine sarcomas. Nagamatsu et al. previously reported that the intratumoral standardized uptake value (SUV) obtained from FDG-PET imaging was useful for the differential diagnosis of leiomyosarcoma and leiomyoma, and that when the cut-off for SUV was set to 3, the rate of diagnostic accuracy was 0.79 (sensitivity, 1.0; specificity, 0.73) (Nagamatsu et al. 2010). Future studies will also be needed to determine whether FDG-PET could be used as a predictor in the preoperative diagnosis of uterine sarcomas.

In the present study, we found endometrial cytology to be a significantly useful evaluation tool. Oda et al. (Oda et al. 2004) suggested that the presence of “spindle or multinucleated giant cells with scanty cytoplasm; relatively large, hyperchromatic nuclei; and conspicuous nucleoli” in endometrial cytology findings may be an indication of uterine sarcoma. However, preoperative diagnosis of uterine sarcoma from endometrial cytology or endometrial biopsy may not be possible without tumor growth into the uterine cavity.

Uterine sarcoma has a very poor prognosis. Tumor stage is the most important prognostic factor for all histologic types, with 5-year overall survival of 50–55% for stage I and 8–12% for stage II–IV disease (Gadducci et al. 2008). Early diagnosis and hysterectomy are key factors for improving prognosis. However, preoperative diagnosis and staging of uterine sarcoma is challenging, and it is extremely difficult to identify advanced cases with distant metastasis.

Early surgery is recommended for suspected cases of uterine sarcoma in order to obtain material for histopathological study. However, few of these patients are actually diagnosed with uterine sarcoma, while most have benign uterine tumors such as myoma. Of our 63 cases that underwent surgery for suspected uterine sarcoma, only 15 (23.8%) were diagnosed with sarcoma, whereas the remaining 48 cases did not require any surgery. In actual practice, then, three-fourths of patients underwent unnecessary surgical procedure.

Prior to our study, no scoring system using multiple predictors has been used for the preoperative diagnosis of uterine sarcoma. We created PRESS by combining multiple predictors for diagnosis of uterine sarcoma. The optimum PRESS cut-off score is 3 points, at which the accuracy rate is 84.1%. In this study, the proposed PRESS seems to have significant benefit for making treatment decisions in a clinical setting for suspected uterine sarcoma cases. Those with 3 or higher points are advised to undergo immediate surgery for diagnosis of the disease. If the score is low, however, surgery may be avoided, and conservative treatment presented to the patient as an informed choice. For example, premenopausal patients with low PRESS scores and tumors that decrease in size after gonadotropin-releasing hormone (GnRH) analog treatment may be presumed to be uterine myomas, and unnecessary surgical procedures can be avoided. Nevertheless, 3 uterine sarcoma cases in our study had a score of 2 or lower points (the score was 2 points in all cases). One of them was a 51-year-old post-menopausal woman who complained of lower abdominal pain. A 9-cm intrauterine tumor was found. The patient’s serum CA125 level was elevated (180 U/mL), but RI was low (0.39). No abnormalities were observed in serum LDH level, MRI findings, or endometrial cytology findings. The final pathological diagnosis after hysterectomy was ESS. Considering low PRESS score in the decision to perform surgery should therefore be interpreted with caution. In addition, a 12-cm uterine tumor was found in a 71-year-old woman with abnormal vaginal bleeding. The intratumoral RI showed a low value (0.25), but the serum LDH level, MRI finding, and endometrial cytology finding showed no abnormality. After hysterectomy, the final pathological diagnosis was leiomyosarcoma. Another 38-year-old premenopausal woman visited our hospital with the chief complaint of abnormal vaginal bleeding and was found to have a 4.9-cm uterine tumor, which was categorized as class III according to endometrial cytology findings. Neither serum LDH level nor MRI finding showed any abnormality. The final pathological diagnosis was adenosarcoma. We would like to emphasize that PRESS is only intended to serve as reference for selecting therapeutic strategies, and that it is not a universal reference.

Our proposed PRESS should be considered a prototype diagnostic score. The PRESS will continue to undergo modifications as more data become available. In the future, a diagnostic scoring system with high accuracy by adding new predictors and considering point distribution needs to be developed.

## Conclusions

Using multiple predictors for preoperatively diagnosing uterine sarcoma, our proposed PRESS scoring system is beneficial in the clinical setting while making treatment decisions in suspected uterine sarcoma cases and thus, avoiding unnecessary surgeries.

## Abbreviations

PRESS: Preoperative Sarcoma Score
MRI: Magnetic resonance imaging
LDH: Lactate dehydrogenase
RI: Resistance index
CA125: Cancer antigen 125
IRB: Institutional Review Board
ROC: Receiver operating characteristic
SD: Standard deviation
ESS: Endometrial stromal sarcoma
PPV: Positive predictive value
NPV: Negative predictive value
FIGO: the International Federation of Gynecology and Obstetrics
Gd-DTPA: Gadolinium Diethylenetriaminepenta-acetic Acid
FDG-PET: Fluorine-18-fluorodeoxyglucose positron emission tomography
SUV: Standardized uptake value
GnRH: Gonadotropin-releasing hormone.
